# A Combined Digital and Biomarker Diagnostic Aid for Mood Disorders (the Delta Trial): Protocol for an Observational Study

**DOI:** 10.2196/18453

**Published:** 2020-08-10

**Authors:** Tony Olmert, Jason D Cooper, Sung Yeon Sarah Han, Giles Barton-Owen, Lynn Farrag, Emily Bell, Lauren V Friend, Sureyya Ozcan, Nitin Rustogi, Rhian L Preece, Pawel Eljasz, Jakub Tomasik, Daniel Cowell, Sabine Bahn

**Affiliations:** 1 Department of Chemical Engineering and Biotechnology University of Cambridge Cambridge United Kingdom; 2 Psyomics Ltd Cambridge United Kingdom

**Keywords:** proteomics, early diagnosis, mood disorders, bipolar disorder, major depressive disorders

## Abstract

**Background:**

Mood disorders affect hundreds of millions of people worldwide, imposing a substantial medical and economic burden. Existing diagnostic methods for mood disorders often result in a delay until accurate diagnosis, exacerbating the challenges of these disorders. Advances in digital tools for psychiatry and understanding the biological basis of mood disorders offer the potential for novel diagnostic methods that facilitate early and accurate diagnosis of patients.

**Objective:**

The Delta Trial was launched to develop an algorithm-based diagnostic aid combining symptom data and proteomic biomarkers to reduce the misdiagnosis of bipolar disorder (BD) as a major depressive disorder (MDD) and achieve more accurate and earlier MDD diagnosis.

**Methods:**

Participants for this ethically approved trial were recruited through the internet, mainly through Facebook advertising. Participants were then screened for eligibility, consented to participate, and completed an adaptive digital questionnaire that was designed and created for the trial on a purpose-built digital platform. A subset of these participants was selected to provide dried blood spot (DBS) samples and undertake a World Health Organization World Mental Health Composite International Diagnostic Interview (CIDI). Inclusion and exclusion criteria were chosen to maximize the safety of a trial population that was both relevant to the trial objectives and generalizable. To provide statistical power and validation sets for the primary and secondary objectives, 840 participants were required to complete the digital questionnaire, submit DBS samples, and undertake a CIDI.

**Results:**

The Delta Trial is now complete. More than 3200 participants completed the digital questionnaire, 924 of whom also submitted DBS samples and a CIDI, whereas a total of 1780 participants completed a 6-month follow-up questionnaire and 1542 completed a 12-month follow-up questionnaire. The analysis of the trial data is now underway.

**Conclusions:**

If a diagnostic aid is able to improve the diagnosis of BD and MDD, it may enable earlier treatment for patients with mood disorders.

**International Registered Report Identifier (IRRID):**

DERR1-10.2196/18453

## Introduction

### Background

Mood disorders affect approximately 400 million people worldwide, with bipolar disorder (BD) and major depressive disorder (MDD) representing the 17th and the 2nd leading causes of years lost to disability, respectively [1]. Affected individuals experience debilitating and often recurrent symptoms [2,3] as well as an association with high levels of both psychiatric and somatic comorbidities [4,5], culminating in decreased quality of life [6,7] and increased mortality [8]. In addition, caregivers of patients with mood disorders experience a substantial burden associated with this role, expanding the direct impact of these conditions [9,10]. Economically, recent estimates of the annual European-wide costs associated with BD are €21.5 billion (US $24.1 billion), whereas those associated with MDD are €91.9 billion (US $102.9 billion) [11].

Existing diagnostic methodology, based on subjective reports and observations gathered during clinical interviews that are referenced against symptom checklists [12], leads to frequent misdiagnosis and underdiagnosis of mood disorders [13,14]. Patients with BD often initially present with depressive episodes that can be indistinguishable from depressive episodes in the context of MDD [15,16], leading to approximately half or more of patients with BD initially being misdiagnosed with MDD [17,18]. The approximate 8- to 10-year delay before a BD diagnosis includes the delay from initial symptom manifestation until psychiatric evaluation as well as the delay from initial assessment until correct diagnosis [19,20]. MDD diagnosis faces issues of both over- and underdiagnosis. These issues overlap with one another and include patient reluctance to seek help for emotional distress [21], short consultation times, and limited focus on mental health in primary care [22]; a shortage of mental health practitioners [23,24]; and the difficulty of identifying patients who fulfill the clinical threshold for MDD [25].

Although pharmacological and psychological treatments can be effective when correctly prescribed [26-28], inaccurate or delayed diagnosis limits the effective use of these treatments. For BD, a delay before correct diagnosis and treatment is associated with poorer outcomes [29], whereas a diagnostic delay for MDD compounds the time associated with the identification of an effective therapy, which can lead to months of trial and error treatment testing [27,30]. By shortening the period before the administration of appropriate treatment, early and accurate diagnosis of mood disorders represents the first step on the path to lessen the burden experienced by patients with BD and MDD.

Psychiatry is increasingly turning to a variety of digital tools, which are already in use in many forms [31], to improve symptom-based diagnosis [32]. One reason for this approach is the unprecedented data gathering opportunities that digital platforms represent for researchers [33]. However, despite the optimistic projections of technology’s potential to increase the accessibility and efficacy of psychiatric interventions, these goals are not yet fully realized [34,35]. With digital solutions in psychiatry still in an early phase of research and development, they may be most immediately clinically applicable in areas with fewer barriers to validation and adoption. One such area is psychiatric questionnaires, for which digitization has already begun. In particular, the increased convenience of digital questionnaires [36] may overcome clinicians’ stated obstacles of time and difficulty of implementation [37]. As the majority of these questionnaires have shown interformat reliability, meaning the digital and paper versions are comparable [38], the next steps for digital questionnaire development may be the personalization of questions to individual patients through dynamic question selection. With the digitization of previously existing psychiatric questionnaires ongoing, digital psychiatric questionnaires represent a promising field for further innovation.

In conjunction with the progression to digital tools to evaluate patient history and symptoms, a paradigm shift to biomarker-based diagnosis in psychiatry is underway [39]. This shift is facilitated by both the growing understanding of the biological basis of mood disorders [40,41] as well as advances in technology that allow large amounts of biological information to be collected and analyzed. Biomarkers are defined by the National Institute of Health’s Biomarker Working Group as “a characteristic that is objectively measured and evaluated as an indicator of normal biological processes, pathogenic processes, or pharmacologic responses to a therapeutic intervention,” with diagnostic biomarkers representing one of a number of categories of biomarkers, alongside prognostic and predictive biomarkers [42]. It is important to note, however, that the clinical utility of diagnostic biomarkers is not dependent on a full understanding of the complex and interrelated factors culminating in psychiatric illness. Despite the growing research into biomarkers, to date, no diagnostic biomarkers for psychiatric disorders have been implemented in routine clinical use. In pursuit of this goal, efforts for biomarker discovery and validation are widespread, supported by initiatives such as the National Institute of Mental Health’s Research Domain Criteria project [43].

Proteomics is a promising area for biomarker development because of its ability to detect disease-related alterations in readily accessible bodily fluids such as blood. This potential has been supported by research on candidate biomarkers and preliminary results both in mood disorders and other psychiatric conditions [44]. However, because of the complexity and heterogeneity of psychiatric disorders [45-47], there is a high threshold for progress in identifying and validating diagnostic psychiatric biomarkers. It is, therefore, likely that a panel of multiple biomarkers, rather than a single one, will be necessary for the validation of a psychiatric diagnostic aid [44]. Although much research on biomarkers has been previously conducted using serum or plasma, dried blood spots (DBS) represent a novel and clinically promising methodology for validating psychiatric biomarkers, given the multiple advantages of DBS that decrease barriers to implementation. These advantages include lower cost, minimal invasiveness, decreased blood volume required, simplified shipping and storage, and the ability of patients to provide a sample in a nonclinical setting [48]. When analyzing DBS samples, the strengths of mass spectrometry (MS), such as the ability to quantify large numbers of analytes in parallel, high sensitivity and specificity, and reproducibility [48,49], make it an ideal technique for validating a psychiatric diagnostic panel. By leveraging existing proteomics research in psychiatry with the complementary strengths of DBS and MS analysis, the goal of validating a biomarker-based diagnostic aid for mood disorders could be achieved.

To validate new approaches and tools to advance the process of psychiatric diagnosis, large-scale diagnostic trials are required [49-51]. However, the difficulty of reaching trial recruitment goals makes recruitment an obstacle to consider in its own right. Although the proportion of research trials reaching their recruitment goals seems to be improving, slightly under half of the publicly funded clinical trials still fail to reach these benchmarks [52-54]. This issue has been specifically documented in mood disorder trials, with multiple trials in recent years having failed because of under-recruitment [55,56]. The substantial time and cost devoted to failed trials [57] and the high proportion of trials that experience recruitment extensions [52] divert resources from clinical objectives. Beyond reaching a target number of trial participants, a representative participant population is required to ensure the generalizability and reproducibility of findings [58]. This is of particular concern in psychiatry, as the generalizability of research trials and clinical usefulness of findings has been questioned and remains under scrutiny [59-62]. In the case of web-based recruitment in psychiatry, it is also unclear how closely a trial population reflects the population of interest, as some groups may face digital exclusion [63]. Executing web-based trial recruitment in a timely and cost-effective manner could provide a roadmap for recruitment for other psychiatric trials.

Diagnostic tools must both accurately identify conditions and encourage appropriate follow-up action to improve treatment and outcomes for patients. Given the difficulty of effective communication between clinicians, academic researchers, and patients in psychiatry [64,65], the inclusion of service users in trial conception, planning, and design is increasingly considered essential to achieve this aim [66]. Specific to diagnostic trials, considering the trial in the broader context of the health care delivery pathway contributes to the clinical effectiveness of the diagnostic tool that is eventually developed [51]. By investigating the relationships between diagnostic information, caregiver decision making, and patient perceptions, the gaps in the mood disorder care pathway may be better understood and narrowed.

This study describes the objectives, methods, and recruitment results for the Delta Trial, which was launched in April 2018 and closed in February 2020 by the University of Cambridge’s Cambridge Centre for Neuropsychiatric Research (CCNR). The aim of the trial is to develop algorithms based on a digital questionnaire and proteomic biomarker data to be used as a diagnostic aid for mood disorders in patients presenting with depressive symptoms. Participants were recruited through the internet and completed the trial remotely in a manner that may provide a useful roadmap for future psychiatric trials. The analysis of these data is now underway. The creation of a diagnostic aid for mood disorders that combines questionnaire and biomarker data could contribute to reducing the significant individual and societal burden of mood disorders by facilitating early diagnosis, leading to effective treatment.

### Objectives

#### Primary Objective: To Reduce the Misdiagnosis of Bipolar Disorder

The primary objective of the Delta Trial is to develop a diagnostic algorithm based on a digital questionnaire and proteomic data to identify individuals with BD who have been misdiagnosed with MDD. The target population for the primary objective was participants who had received a recent MDD diagnosis from a general practitioner or psychiatrist and were experiencing some depressive symptoms at the time of recruitment (baseline MDD). *Recent* was defined as a diagnosis within the last 5 years, during which time it would be more likely that a patient with BD remains misdiagnosed with MDD [67,68]. Depressive symptoms were measured using the Patient Health Questionnaire (PHQ-9) [69], with a score of 5 or greater required for inclusion in the trial.

#### Secondary Objective: To Achieve a More Accurate and Earlier Diagnosis of Major Depressive Disorder

The secondary objective of the Delta Trial is to develop a diagnostic algorithm, also based on a digital questionnaire and proteomic data, to identify symptomatic help seekers with MDD. The target population for the secondary objective was participants who had not had a previous mood disorder diagnosis (baseline low mood) and scored 5 or greater on the PHQ-9 at the time of recruitment. Diagnoses predicted by the algorithms developed for the primary and secondary objectives will be compared with the diagnoses obtained through the World Health Organization World Mental Health Composite International Diagnostic Interview (CIDI), which is assumed to represent the participant’s true diagnosis [70].

#### Follow-Up Objectives

Follow-up objectives for investigating participants’ response following the receipt of a results report were also established to understand the response to, and effectiveness of, a diagnostic aid such as that under investigation in the Delta Trial within existing mental health care pathways. These follow-up objectives, informed by data from digital 6- and 12-month follow-up questionnaires, are to understand whether trial participation (1) impacts the quality of life for participants, as measured by the Warwick-Edinburgh Mental Wellbeing Scale [71]; (2) leads to new or changed mood disorder diagnoses; or (3) results in a recommendation of new or changed mood disorder–related medications and interventions.

#### Statistical Calculations

We assumed that we could detect at least 80% of both participants with BD who had a baseline MDD diagnosis and of baseline low mood participants who had undiagnosed MDD. In addition, based on previous studies [72-75], we assumed that 20% or more of participants in the trial with a baseline MDD diagnosis would have BD and that 30% or more of the baseline low mood participants would have MDD. Power calculations estimated that we would need to recruit at least 200 participants for each of the primary and secondary objectives to have more than 80% power to detect noninferiority to a prediction model with an area under the receiver operating characteristic curve of 0.8 at the 5% significance level. To account for participant attrition and other factors, we decided to recruit 300 participants for each of the primary and secondary objectives to form the training sets for algorithm development. To provide test sets to validate the algorithms to be developed, a further 100 participants for each objective as well as 40 participants with a baseline BD diagnosis (baseline BD) who scored 5 or greater on the PHQ-9 at the time of recruitment were added to the data collection targets. The recruitment for these groups was also stratified to reflect the observed gender ratios of the relevant conditions: two-thirds women for the baseline MDD and low mood groups and evenly split between men and women for the baseline BD group [76].

## Methods

### Recruitment

Participant recruitment for the Delta Trial was executed through emails to suitable participants from previous trials who had consented to be recontacted, paid Facebook advertisements, organic and paid promoted posts on the CCNR Facebook page [77], and updates to the CCNR laboratory website [78]. The choice of exclusively using Facebook for paid advertising was based on experience from a previous pilot study (unpublished), demonstrating its superior effectiveness over other digital advertising options. Both static imagery and animated videos were created and used as advertising materials for the trial.

### Eligibility Screening, Consent, and Enrollment

Inclusion criteria and consent requirements for participants are listed in Table 1. These criteria were kept to a minimum within the bounds of safety and resource constraints to ensure that representative samples from the target populations were recruited. Participants were able to complete an eligibility screening, including the PHQ-9, and were provided with a participant information leaflet as well as the opportunity to ask questions about the trial before consenting to participate through the Delta Trial website [79]. To maximize the safety of those individuals who were interested in participating in the Delta Trial but were not eligible because of current suicidal ideation, we provided specific resources and contact information when they felt unsafe. Eligible participants who provided consent were sent a confirmation email to ensure that they had registered with an email address to which they had regular access. Acknowledgment of receipt of this email was considered the final step of enrollment in the trial.

**Table 1:** Delta Trial inclusion criteria and consent process.

Participants are eligible to perform	Inclusion criteria or actions required
Adaptive digital questionnaire (selection through eligibility screening, consent, and enrollment)	Age 18-45 years UK resident Not pregnant or breastfeeding Not suicidal Patient Health Questionnaire 9 score ≥5 Consent to having read participant information sheet Consent to voluntary participation Select link in the confirmation email
DBS^a^ and CIDI^b^ (selection through consent, digital questionnaire responses, and internal analysis; only eligible once the digital questionnaire is completed)	Consent to provide DBS samples and complete CIDI No blood-borne illness No previous diagnosis of schizophrenia Recruiting target not yet reached for baseline mood disorder diagnosis group (major depressive disorder, bipolar disorder, or low mood^c^)

^a^DBS: dried blood spot.

^b^CIDI: Composite International Diagnostic Interview.

^c^Low mood: no mood disorder diagnosis.

### Delta Trial Digital Platform

Once participants were enrolled in the trial, they were able to access the Delta Trial digital platform by logging on to the Delta Trial website. The Delta Trial digital platform incorporated input from a service user advisory group on features such as the tone of written materials, frequency of communication, and participant journey through the platform and was developed under Medical Device Directive 93/42/EEC. An individualized dashboard on this web platform guided each participant’s progress through the trial. All digitized steps of the trial were completed through this platform, which automatically recorded and visualized every step through the trial for participants. The Delta Trial digital platform was designed for ease of use to encourage completion of the trial, and specific functionality of the digital platform that facilitated this goal included automated reminder emails for each step of the trial that were sent to participants at different intervals, the ability to stop and later restart the adaptive questionnaire after any question, and the option to change from one device to another at any time.

### Adaptive Digital Questionnaire

Once enrolled, the first step for all participants in the trial was to complete an adaptive digital questionnaire on the Delta Trial digital platform. A novel adaptive digital questionnaire was created for the Delta Trial. This questionnaire was designed following an analysis of existing questionnaires for mood disorders, the *Diagnostic and Statistical Manual of Mental Disorders,* Fifth Edition [80], and the *International Classification of Diseases and Related Health Problems,* Tenth Revision [81], as well as input from psychiatrists and a service user group. A wide range of studies and questionnaires were analyzed [70,82-90] to ensure the inclusion of well-validated symptoms of BD and MDD, symptoms to discriminate conditions between and within the BD and MDD spectra, and further symptoms and lifestyle factors of potential interest for analysis. The 6 sections of the questionnaire focused on the following topics: (1) demographic information and personal history; (2) manic and hypomanic symptoms; (3) depressive symptoms; (4) personality profiling; (5) medication, treatment, and substance use history; and (6) other psychiatric conditions. Each section of the questionnaire was estimated to require an average of 12 (SD 3) min to complete, although actual times varied because of the adaptive nature of the questionnaire, which directed participants to relevant questions based on their previous answers. The questionnaire database contained a total of 635 distinct questions, with the longest possible route through a questionnaire consisting of 382 questions.

After completion of the digital questionnaire, participants were either asked to provide DBS samples or were marked to receive a results report. This decision was made through a two-step process: first requiring consent from participants during enrollment that they were willing to perform DBS sampling and a telephone interview, followed by selection based on the fulfillment of the second set of inclusion criteria, as listed in Table 1. Participants who had not consented to do so or were not eligible to provide DBS samples and perform a telephone interview were delivered a nondiagnostic results report through the digital platform that described the most likely conditions according to their answers to the digital questionnaire.

### Dried Blood Spot Collection for Proteomic Analysis

Participants selected to continue with the trial were asked to input an address through the digital platform, to be sent a DBS sample collection kit. The DBS sample collection kit used in the Delta Trial was a Conformité Européene-marked device under Article 22 of the Medical Device Regulation 2017/745 and contained relevant materials and instructions to allow participants to complete and submit DBS samples.

A standardized MS-based targeted proteomic biomarker screening method, based on previously published work [48,91], was developed for the Delta Trial. For this method, 203 candidate peptides representing 120 proteins were selected for inclusion, in many cases based on their previous association with psychiatric disorders, including depression, BD, and schizophrenia [91]. These proteins were first extracted and digested from the DBS samples [48], and then, unique surrogate peptides representing candidate proteins were monitored through multiple reaction monitoring using an Agilent 1290 liquid chromatography system coupled with an Agilent 6495 Triple Quadrupole Mass Spectrometer.

### Composite International Diagnostic Interview

For the Delta Trial, predictions from the algorithms developed for the primary and secondary objectives will be compared with the result of a CIDI, which is assumed to represent the participant’s diagnosis [70]. These interviews were conducted over the telephone and using the CIDI 3.0 software version, developed by the World Health Organization. For these interviews, the only sections of the CIDI software that were operationalized were the screening section questions related to demographics or mood disorders as well as the depression and mania sections. All interviewers received in-person external training from a CIDI-certified instructor as well as internal training and monitoring. After completion of the CIDI, participants received results reports similar to those received by participants who only completed the digital questionnaire. The reports for these participants were identical to those received by participants who only completed the questionnaire, except that these reports reflected the mood disorder condition suggested by the CIDI. Only participants who completed all 3 steps—comprising a completed digital questionnaire, DBS samples, and CIDI—fulfilled the recruitment targets from the statistical calculations.

### Feedback

Following receipt of a results report, all participants were asked to complete a short feedback survey through the digital platform. This survey was intended to guide future trial design and gain insight into participant perceptions related to the trial.

### Follow-Up

The final step in the trial is follow-up questionnaires to be completed through the digital platform at 6 and 12 months from the date that a participant received a results report. These questionnaires were designed to provide answers via patient self-reports to the follow-up objectives related to the impact of Delta Trial participation. These objectives are to analyze and understand whether trial participation (1) impacts the quality of life for participants, (2) leads to new or changed mood disorder diagnoses, or (3) results in a recommendation of new or changed mood disorder–related medications and interventions.

### Ethics and Data Handling

All trial-related materials and methods were ethically approved by the University of Cambridge Human Biology Research Ethics Committee (approval number HBREC 2017.11) and conducted in accordance with Good Clinical Practice and International Organization for Standardization (ISO 14155:2011). All participants in the Delta Trial had access to a downloadable participant information sheet as well as other information related to the trial and general psychoeducation via the Delta Trial website and were emailed their digitally signed and dated consent forms and the participant information sheet on enrollment in the trial. All trial data are stored securely at the University of Cambridge’s CCNR, and confidentiality is maintained using unique participant ID numbers and digitally separating trial data from participants’ personal information. Personal information, which is only accessible by a selected number of trial staff, is kept solely for the purposes of recontacting participants who have opted in for future CCNR studies or as dictated by the General Data Protection Regulation and Human Tissue Authority and will not be shared with any other organizations.

## Results

### Timeline

The recruitment for the Delta Trial was launched on April 27, 2018, and closed on September 28, 2018. All participants who returned DBS samples before October 16, 2018, were asked to perform a CIDI, and the final CIDI was conducted on October 24, 2018. Outstanding results reports for participants who had progressed to the end of the digital questionnaire were delivered on October 26, 2018. Feedback on trial processes and perceptions of the trial was submitted by 1289 participants following receipt of a results report. A total of 1780 participants also completed a 6-month follow-up questionnaire and 1542 completed a 12-month follow-up questionnaire, the last of which was completed in November 2019. These participants did not necessarily overlap with those who provided DBS samples and completed a CIDI. The trial was officially closed on February 6, 2020.

### Recruitment Results

**Figure 1:**
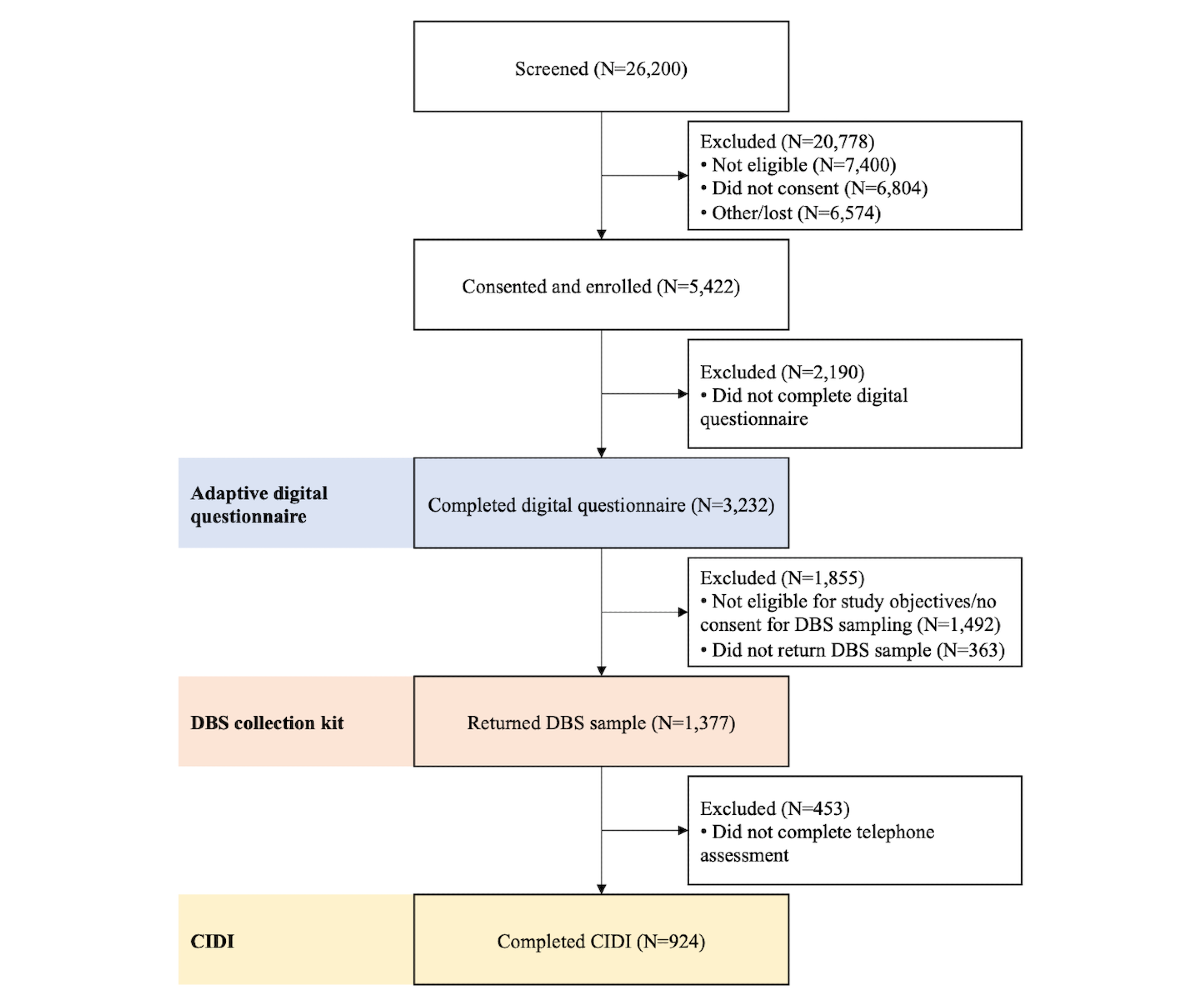
Delta Trial flow chart. This figure illustrates the number of individuals who completed each step of the Delta Trial and the reasons for attrition between each step. Abbreviations: DBS: dried blood spot; CIDI: World Health Organization World Mental Health Composite International Diagnostic Interview.

To achieve training and validation datasets for the diagnostic algorithms as dictated by the power calculations for the primary and secondary objectives, 5422 participants were enrolled in the trial. The observed progress of the participants is documented in Figure 1 and Table 2. The results reports were delivered to 3232 participants who completed the digital questionnaire, and an average of 284 questions were answered by each participant who completed the questionnaire. One step of the trial for which the participant completion rate was notable was in the return of DBS sample collection kits, as 79.14% (1377/1740) of kits that were posted to participants were completed and returned to CCNR. In addition, 962 participants with a diagnosis of MDD that was established greater than 5 years before enrollment but otherwise fulfilled enrollment criteria were enrolled and completed the digital questionnaire. These participants did not progress to further stages of the trial.

**Table 2:** Delta Trial participant progress, grouped by baseline mood disorder diagnosis group (N=5422).

Mood disorder diagnosis group		Trial steps and overall completion rate from previous step, n (%)			
		Completed digital questionnaire 3232 (59.61)	Sent DBS^a^ sample collection kit 1740 (53.84)	Returned DBS sample collection kit 1377 (79.14)	Performed Composite International Diagnostic Interview 924 (67.10)
**Major depressive disorder, n (%)**					
	Female	676 (71.9)	610 (71.3)	467 (69.7)	301 (68.4)
	Male	264 (28.1)	246 (28.7)	203 (30.3)	139 (31.6)
**Bipolar disorder, n (%)**					
	Female	144 (64.0)	26 (34)	23 (34)	21 (39)
	Male	81 (36.0)	50 (66)	45 (66)	33 (61)
**Low mood^b^, n (%)**					
	Female	668 (68.58)	534 (66.6)	411 (64.8)	277 (64.6)
	Male	306 (31.42)	268 (33.4)	223 (35.2)	152 (35.4)
**Other^c,d^, n (%)**					
	Female	837 (76.58)	5 (83)	4 (80)	1 (100)
	Male	256 (23.42)	1 (17)	1 (20)	0 (0)

^a^DBS: dried blood spot.

^b^Low mood: no mood disorder diagnosis.

^c^Other: not in one of the major depressive disorder, bipolar disorder, or low mood baseline mood disorder diagnosis groups.

^d^Participants from this group were able to progress beyond the digital questionnaire because of operator error in the trial progression selection process.

A completed digital questionnaire, DBS samples, and a CIDI were gathered from 924 participants. There were 440 participants in the baseline MDD group, 54 participants in the baseline BD group, and 429 participants in the baseline low mood group. The gender ratios of these groups also generally reflected pretrial targets, as the percentage of female participants in the groups was 68.4% for baseline MDD, 39% for baseline BD, and 64.6% for baseline low mood. Any noteworthy differences in group characteristics will be accounted for in future statistical analyses.

## Discussion

### Principal Findings

The Delta Trial was designed and launched with the ultimate aim of contributing to early and accurate diagnosis of mood disorders to enable effective treatment for patients. Specifically, the objectives of the trial are to develop and validate diagnostic algorithms to reduce the misdiagnosis of BD as MDD and achieve more accurate and earlier diagnosis of MDD in a population of participants with depressive symptoms who completed the trial remotely. To this end, processes and techniques that can be implemented in a real-world setting were established, such as the creation of a novel adaptive digital questionnaire and the use of self-collected DBS samples for proteomic analysis. More participants than were dictated by data collection targets for the primary and secondary objectives were recruited and enrolled in the trial over the course of 5 months. In addition, the trial population approximately matched baseline mood disorder diagnosis groups and the level of gender stratification that were targeted before launching the trial. These successes are emblematic of the opportunities that are possible through leveraging a combination of digital tools and a consideration of the viewpoints of multiple stakeholders, including a service user group and clinicians.

### Strengths and Limitations

However, the design and execution of the Delta Trial also introduced potential bias in the participant population, most notably through (1) recruitment techniques, (2) the use of self-reports of medical history, and (3) the selection of participants to progress through the various stages of the trial. With recruitment conducted on the web and mainly through Facebook, the participants in the trial are likely to be more regular internet—and specifically Facebook—users than the target population. In addition, variables such as age, gender, and interests used in displaying advertising materials may have shaped the composition of participants according to the recruitment targets described earlier. Self-reports of medical history for participants and their families may have been incorrect or incomplete and could have been improved by using medical records. In addition, the progressive nature of the trial, in which participants had to consent for the DBS and CIDI steps of the trial and were then selected to perform those steps, shaped the trial population to further conform to the recruitment targets and population assumptions underlying those targets. Finally, the time commitment required to complete the questionnaire, submit DBS samples, and perform a CIDI required a level of interest in the trial that may have been higher in people who were very concerned about their mental health symptoms or history, enriching the levels of undiagnosed conditions in the trial population.

### Conclusions

The Delta Trial was launched in April 2018 with the aim of creating a diagnostic aid to improve the diagnosis of mood disorders. Demographic and symptom data have been gathered from 3232 participants who completed an adaptive digital questionnaire that was designed and created for the trial. Of these participants, 924 also submitted DBS samples for proteomic analysis and completed a CIDI. With follow-up questionnaires completed in November 2019 and the trial closed in February 2020, the analysis of the trial data has now begun. We hope to share our results to contribute to reducing the significant burden of mood disorders through early and accurate diagnosis. We also hope to gain insight into follow-up actions taken by participants after receiving a results report and the process of digital recruitment for psychiatric trials.

## Abbreviations

BD: bipolar disorder
CCNR: Cambridge Centre for Neuropsychiatric Research
CIDI: Composite International Diagnostic Interview
DBS: dried blood spot
MDD: major depressive disorder
MS: mass spectrometry
PHQ-9: Patient Health Questionnaire

## References

[1] Global Burden of Disease Study 2013 Collaborators (2015). Global, regional, and national incidence, prevalence, and years lived with disability for 301 acute and chronic diseases and injuries in 188 countries, 1990-2013: a systematic analysis for the global burden of disease study 2013. Lancet.

[2] Kessler RC (2012). The costs of depression. Psychiatr Clin North Am.

[3] Miller S, Dell'Osso B, Ketter T (2014). The prevalence and burden of bipolar depression. J Affect Disord.

[4] Gadermann A, Alonso J, Vilagut G, Zaslavsky AM, Kessler RC (2012). Comorbidity and disease burden in the national comorbidity survey replication (NCS-R). Depress Anxiety.

[5] Magalhães PV, Kapczinski F, Nierenberg AA, Deckersbach T, Weisinger D, Dodd S, Berk M (2012). Illness burden and medical comorbidity in the systematic treatment enhancement program for bipolar disorder. Acta Psychiatr Scand.

[6] Alonso J, Angermeyer M, Lépine JP, European Study of the Epidemiology of Mental Disorders (ESEMeD) Project (2004). The European study of the epidemiology of mental disorders (ESEMeD) project: an epidemiological basis for informing mental health policies in Europe. Acta Psychiatr Scand Suppl.

[7] Michalak E, Yatham L, Lam RW (2005). Quality of life in bipolar disorder: a review of the literature. Health Qual Life Outcomes.

[8] Chesney E, Goodwin G, Fazel S (2014). Risks of all-cause and suicide mortality in mental disorders: a meta-review. World Psychiatry.

[9] Ostacher MJ, Nierenberg AA, Iosifescu DV, Eidelman P, Lund HG, Ametrano RM, Kaczynski R, Calabrese J, Miklowitz DJ, Sachs GS, Perlick DA, STEP-BD Family Experience Collaborative Study Group (2008). Correlates of subjective and objective burden among caregivers of patients with bipolar disorder. Acta Psychiatr Scand.

[10] van Wijngaarden B, Schene A, Koeter MW (2004). Family caregiving in depression: impact on caregivers' daily life, distress, and help seeking. J Affect Disord.

[11] Olesen J, Gustavsson A, Svensson M, Wittchen HU, Jönsson B, CDBE 2010 Study Group, European Brain Council (2012). The economic cost of brain disorders in Europe. Eur J Neurol.

[12] Andreasen NC (2007). DSM and the death of phenomenology in America: an example of unintended consequences. Schizophr Bull.

[13] Tyrer P (2009). Are general practitioners really unable to diagnose depression?. Lancet.

[14] van Weel-Baumgarten E, Lucassen P (2009). Clinical diagnosis of depression in primary care. Lancet.

[15] Hirschfeld R (2014). Differential diagnosis of bipolar disorder and major depressive disorder. J Affect Disord.

[16] Frankland A, Cerrillo E, Hadzi-Pavlovic D, Roberts G, Wright A, Loo Ck, Breakspear M, Mitchell PB (2015). Comparing the phenomenology of depressive episodes in bipolar I and II disorder and major depressive disorder within bipolar disorder pedigrees. J Clin Psychiatry.

[17] Ghaemi S, Sachs GS, Chiou AM, Pandurangi AK, Goodwin FK (1999). Is bipolar disorder still underdiagnosed? Are antidepressants overutilized?. J Affect Disord.

[18] Hirschfeld R, Lewis L, Vornik LA (2003). Perceptions and impact of bipolar disorder: how far have we really come? Results of the national depressive and manic-depressive association 2000 survey of individuals with bipolar disorder. J Clin Psychiatry.

[19] Bauer M, Andreassen O, Geddes JR, Kessing LV, Lewitzka U, Schulze TG, Vieta E (2018). Areas of uncertainties and unmet needs in bipolar disorders: clinical and research perspectives. Lancet Psychiatry.

[20] Patel R, Shetty H, Jackson R, Broadbent M, Stewart R, Boydell J, McGuire P, Taylor M (2015). Delays before diagnosis and initiation of treatment in patients presenting to mental health services with bipolar disorder. PLoS One.

[21] Walters K, Buszewicz M, Weich S, King M (2008). Help-seeking preferences for psychological distress in primary care: effect of current mental state. Br J Gen Pract.

[22] Tylee A, Gandhi P (2005). The importance of somatic symptoms in depression in primary care. Prim Care Companion J Clin Psychiatry.

[23] Kakuma R, Minas H, van Ginneken N, dal Poz MR, Desiraju K, Morris JE, Saxena S, Scheffler RM (2011). Human resources for mental health care: current situation and strategies for action. Lancet.

[24] (2017). Our Workforce Census. Royal College of Psychiatrists.

[25] Mitchell A, Vaze A, Rao S (2009). Clinical diagnosis of depression in primary care: a meta-analysis. Lancet.

[26] Bowden C, Perlis R, Thase ME, Ketter TA, Ostacher MM, Calabrese JR, Reilly-Harrington NA, Gonzalez JM, Singh V, Nierenberg AA, Sachs GS (2012). Aims and results of the NIMH systematic treatment enhancement program for bipolar disorder (STEP-BD). CNS Neurosci Ther.

[27] Rush A, Warden D, Wisniewski SR, Fava M, Trivedi MH, Gaynes BN, Nierenberg AA (2009). STAR*D: revising conventional wisdom. CNS Drugs.

[28] Thase ME (2007). STEP-BD and bipolar depression: what have we learned?. Curr Psychiatry Rep.

[29] Franchini L, Zanardi R, Smeraldi E, Gasperini M (1999). Early onset of lithium prophylaxis as a predictor of good long-term outcome. Eur Arch Psychiatry Clin Neurosci.

[30] Leuchter A, Cook I, Hunter A, Korb AS (2009). A new paradigm for the prediction of antidepressant treatment response. Dialogues Clin Neurosci.

[31] Koh S, Cattell G, Cochran DM, Krasner A, Langheim FJ, Sasso DA (2013). Psychiatrists' use of electronic communication and social media and a proposed framework for future guidelines. J Psychiatr Pract.

[32] Patel S, Saunders KE (2018). Apps and wearables in the monitoring of mental health disorders. Br J Hosp Med (Lond).

[33] Torous J, Staples P, Onnela JP (2015). Realizing the potential of mobile mental health: new methods for new data in psychiatry. Curr Psychiatry Rep.

[34] Chan S, Godwin H, Gonzalez A, Yellowlees PM, Hilty DM (2017). Review of use and integration of mobile apps into psychiatric treatments. Curr Psychiatry Rep.

[35] Firth J, Torous J, Nicholas J, Carney R, Pratap A, Rosenbaum S, Sarris J (2017). The efficacy of smartphone-based mental health interventions for depressive symptoms: a meta-analysis of randomized controlled trials. World Psychiatry.

[36] Bauer A, Baldwin S, Anguera JA, Areán PA, Atkins DC (2018). Comparing approaches to mobile depression assessment for measurement-based care: prospective study. J Med Internet Res.

[37] Hatfield D, Ogles BM (2007). Why some clinicians use outcome measures and others do not. Adm Policy Ment Health.

[38] Alfonsson S, Maathz P, Hursti T (2014). Interformat reliability of digital psychiatric self-report questionnaires: a systematic review. J Med Internet Res.

[39] Fernandes BS, Williams LM, Steiner J, Leboyer M, Carvalho AF, Berk M (2017). The new field of 'precision psychiatry'. BMC Med.

[40] Iwata M, Ota K, Duman RS (2013). The inflammasome: pathways linking psychological stress, depression, and systemic illnesses. Brain Behav Immun.

[41] Leboyer M, Soreca I, Scott J, Frye M, Henry C, Tamouza R, Kupfer DJ (2012). Can bipolar disorder be viewed as a multi-system inflammatory disease?. J Affect Disord.

[42] Biomarkers Definitions Working Group (2001). Biomarkers and surrogate endpoints: preferred definitions and conceptual framework. Clin Pharmacol Ther.

[43] Insel T, Cuthbert B, Garvey M, Heinssen R, Pine DS, Quinn K, Sanislow C, Wang P (2010). Research domain criteria (RDoC): toward a new classification framework for research on mental disorders. Am J Psychiatry.

[44] Chan M, Gottschalk M, Haenisch F, Tomasik J, Ruland T, Rahmoune H, Guest PC, Bahn S (2014). Applications of blood-based protein biomarker strategies in the study of psychiatric disorders. Prog Neurobiol.

[45] Wardenaar K, de Jonge P (2013). Diagnostic heterogeneity in psychiatry: towards an empirical solution. BMC Med.

[46] Fried E, Nesse RM (2015). Depression is not a consistent syndrome: an investigation of unique symptom patterns in the STAR*D study. J Affect Disord.

[47] Schmidt H, Shelton R, Duman RS (2011). Functional biomarkers of depression: diagnosis, treatment, and pathophysiology. Neuropsychopharmacology.

[48] Ozcan S, Cooper J, Lago SG, Kenny D, Rustogi N, Stocki P, Bahn S (2017). Towards reproducible MRM based biomarker discovery using dried blood spots. Sci Rep.

[49] Preece R, Han S, Bahn S (2018). Proteomic approaches to identify blood-based biomarkers for depression and bipolar disorders. Expert Rev Proteomics.

[50] Farah M, Gillihan SJ (2012). The puzzle of neuroimaging and psychiatric diagnosis: technology and nosology in an evolving discipline. AJOB Neurosci.

[51] di Ruffano LF, Hyde C, McCaffery KJ, Bossuyt PM, Deeks JJ (2012). Assessing the value of diagnostic tests: a framework for designing and evaluating trials. Br Med J.

[52] Sully B, Julious S, Nicholl J (2013). A reinvestigation of recruitment to randomised, controlled, multicenter trials: a review of trials funded by two UK funding agencies. Trials.

[53] McDonald A, Knight R, Campbell MK, Entwistle VA, Grant AM, Cook JA, Elbourne DR, Francis D, Garcia J, Roberts I, Snowdon C (2006). What influences recruitment to randomised controlled trials? A review of trials funded by two UK funding agencies. Trials.

[54] Walters S, Henriques-Cadby I, Bortolami O, Flight L, Hind D, Jacques RM, Knox C, Nadin B, Rothwell J, Surtees M, Julious SA (2017). Recruitment and retention of participants in randomised controlled trials: a review of trials funded and published by the United Kingdom health technology assessment programme. BMJ Open.

[55] Woodford J, Farrand P, Bessant M, Williams C (2011). Recruitment into a guided internet based CBT (iCBT) intervention for depression: lesson learnt from the failure of a prevalence recruitment strategy. Contemp Clin Trials.

[56] Rendell J, Licht RW (2007). Under-recruitment of patients for clinical trials: an illustrative example of a failed study. Acta Psychiatr Scand.

[57] Kitterman D, Cheng S, Dilts D, Orwoll ES (2011). The prevalence and economic impact of low-enrolling clinical studies at an academic medical center. Acad Med.

[58] Eggener S (2017). Generalizability of clinical trials: why it matters for patients and public policy. Eur Urol.

[59] von Wolff A, Jansen M, Hölzel LP, Westphal A, Härter M, Kriston L (2014). Generalizability of findings from efficacy trials for chronic depression: an analysis of eligibility criteria. Psychiatr Serv.

[60] van der Lem R, van der Wee N, van Veen T, Zitman FG (2011). The generalizability of antidepressant efficacy trials to routine psychiatric out-patient practice. Psychol Med.

[61] Humphreys K, Williams LM (2018). What can treatment research offer general practice?. Lancet Psychiatry.

[62] Wong J, Jones N, Timko C, Humphreys K (2018). Exclusion criteria and generalizability in bipolar disorder treatment trials. Contemp Clin Trials Commun.

[63] Robotham D, Satkunanathan S, Doughty L, Wykes T (2016). Do We Still Have a Digital Divide in Mental Health? A Five-Year Survey Follow-up. J Med Internet Res.

[64] March J, Silva S, Compton S, Shapiro M, Califf R, Krishnan R (2005). The case for practical clinical trials in psychiatry. Am J Psychiatry.

[65] Ditton-Phare P, Halpin S, Sandhu H, Kelly B, Vamos M, Outram S, Bylund CL, Levin T, Kissane D, Cohen M, Loughland C (2015). Communication skills in psychiatry training. Australas Psychiatry.

[66] (2018). National Standards for Public Involvement in Research. INVOLVE.

[67] Berk M, Dodd S, Callaly P, Berk L, Fitzgerald P, de Castella AR, Filia S, Filia K, Tahtalian S, Biffin F, Kelin K, Smith M, Montgomery W, Kulkarni J (2007). History of illness prior to a diagnosis of bipolar disorder or schizoaffective disorder. J Affect Disord.

[68] McIntyre R, Calabrese JR (2019). Bipolar depression: the clinical characteristics and unmet needs of a complex disorder. Curr Med Res Opin.

[69] Kroenke K, Spitzer R, Williams JB (2001). The PHQ-9: validity of a brief depression severity measure. J Gen Intern Med.

[70] Kessler R, Ustün TB (2004). The world mental health (WMH) survey initiative version of the world health organization (WHO) composite international diagnostic interview (CIDI). Int J Methods Psychiatr Res.

[71] Tennant R, Hiller L, Fishwick R, Platt S, Joseph S, Weich S, Parkinson J, Secker J, Stewart-Brown S (2007). The Warwick-Edinburgh mental well-being scale (WEMWBS): development and UK validation. Health Qual Life Outcomes.

[72] Hantouche E, Akiskal H, Lancrenon S, Allilaire J, Sechter D, Azorin J, Bourgeois M, Fraud J, Châtenet-Duchêne L (1998). Systematic clinical methodology for validating bipolar-II disorder: data in mid-stream from a French national multi-site study (EPIDEP). J Affect Disord.

[73] Zuithoff N, Vergouwe Y, King M, Nazareth I, van Wezep MJ, Moons KG, Geerlings MI (2010). The patient health questionnaire-9 for detection of major depressive disorder in primary care: consequences of current thresholds in a crosssectional study. BMC Fam Pract.

[74] Sung S, Low C, Fung D, Chan YH (2013). Screening for major and minor depression in a multiethnic sample of Asian primary care patients: a comparison of the nine-item Patient Health Questionnaire (PHQ-9) and the 16-item quick inventory of depressive symptomatology - self-report (QIDS-SR16 ). Asia Pac Psychiatry.

[75] Benazzi F (1997). Prevalence of bipolar II disorder in outpatient depression: a 203-case study in private practice. J Affect Disord.

[76] (2001). Gender Disparities in Mental Health. World Health Organization.

[77] Cambridge Centre for Neuropsychiatric Research. Facebook.

[78] Cambridge Centre for Neuropsychiatric Research.

[79] The Delta Trial.

[80] American Psychiatric Association (2013). -. Diagnostic and Statistical Manual of Mental Disorders. Fifth Edition.

[81] World Health Organization (1993). ICD-10 Classification of Mental and Behavioural Disorders (The): Diagnostic Criteria for Research.

[82] Hirschfeld RM (2002). The mood disorder questionnaire: a simple, patient-rated screening instrument for bipolar disorder. J Clin Psychiatry.

[83] Eckblad M, Chapman LJ (1986). Development and validation of a scale for hypomanic personality. J Abnorm Psychol.

[84] Ghaemi SN, Miller C, Berv DA, Klugman J, Rosenquist KJ, Pies RW (2005). Sensitivity and specificity of a new bipolar spectrum diagnostic scale. J Affect Disord.

[85] Angst J, Adolfsson R, Benazzi F, Gamma A, Hantouche E, Meyer TD, Skeppar P, Vieta E, Scott J (2005). The HCL-32: towards a self-assessment tool for hypomanic symptoms in outpatients. J Affect Disord.

[86] Leung C, Yim C, Yan CT, Chan CC, Xiang YT, Mak AD, Fok ML, Ungvari GS (2016). The bipolar II depression questionnaire: a self-report tool for detecting bipolar II depression. PLoS One.

[87] Depue R, Slater J, Wolfstetter-Kausch H, Klein D, Goplerud E, Farr D (1981). A behavioral paradigm for identifying persons at risk for bipolar depressive disorder: a conceptual framework and five validation studies. J Abnorm Psychol.

[88] Altman E, Hedeker D, Peterson J, Davis JM (1997). The Altman self-rating mania scale. Biol Psychiatry.

[89] First M, Williams J, Spitzer R, Gibbon M (2007). Structured Clinical Interview for DSM-IV Axis I Disorders (SCID). ePROVIDE - Mapi Research Trust.

[90] Sheehan DV, Lecrubier Y, Sheehan KH, Amorim P, Janavs J, Weiller E, Hergueta T, Baker R, Dunbar GC (1998). The mini-international neuropsychiatric interview (MINI): the development and validation of a structured diagnostic psychiatric interview for DSM-IV and ICD-10. J Clin Psychiatry.

[91] Cooper JD, Han SY, Tomasik J, Ozcan S, Rustogi N, van Beveren NJ, Leweke FM, Bahn S (2019). Multimodel inference for biomarker development: an application to schizophrenia. Transl Psychiatry.

